# Analyzing the influential factors of process safety culture by hybrid hidden content analysis and fuzzy DEMATEL

**DOI:** 10.1038/s41598-024-52067-7

**Published:** 2024-01-17

**Authors:** Mohammad Ghorbani, Hossein Ebrahimi, Shahram Vosoughi, Davoud Eskandari, Saber Moradi Hanifi, Hassan Mandali

**Affiliations:** 1 Occupational Health Research Center, Department of Occupational Health and Safety Engineering, Faculty of Public Health, Iran University of Medical Sciences, Tehran, Iran; 2 Department of Occupational Health and Safety Engineering, School of Health, Shahrekord University of Medical Sciences, Shahrekord, Iran

**Keywords:** Chemical engineering, Health occupations

## Abstract

Due to the complex nature of safety culture and process industries, several factors influence process safety culture. This paper presents a novel framework that combines the hidden content analysis method with Decision Making Trial and Evaluation Laboratory (DEMATEL) and Fuzzy logic to achieve a comprehensive set of influential factors and their relationship. The proposed methodology consists of two primary stages. Firstly, combined methods of literature review and Delphi study were used to identifying influential factors of process safety culture. Secondly, the Fuzzy-DEMATEL approach is employed to quantify and determine the relationships between different influential factors. A diverse pool of experts’ opinions is leveraged to assess the impact of each factor on others and process safety culture. In the first stage, 18 factors identified as influential factors on process safety. The findings of second stage revealed that eight variables were identified as causes, while ten variables were classified as effects. Also, the Organization management's commitment to safety factor had the greatest influence among all of the factors. As well as, the most significant interaction was associated with the risk assessment and management aspect. The integrated approach not only identified the influential factors, but also elucidates the cause-effect relationships among factors. By prioritizing factors and understanding their interconnections, organizations can implement targeted safety measures to improve process safety culture. Its effectiveness in quantifying qualitative data, identifying influential factors, and establishing cause-effect relationships make it a valuable tool for enhancing safety culture in process industries.

## Introduction

Safety culture is an abstract idea that involves integrating individual and group perceptions, thought processes, emotions, and behaviors, which ultimately results in a specific approach to performing tasks within an organization. Safety culture is considered a subset of the overall organizational culture. It encompasses attitudes, beliefs, and perceptions that are acknowledged as standards and values among natural groups, determining their actions in response to hazards and risk control systems^1^. The safety culture is a critical element of an organizational culture, which has a direct impact on the attitudes and behaviors that are related to managing risks either by increasing or decreasing them^2^. This aspect creates personal responsibilities for individuals within the organization and human resource characteristics such as training, development, and adaptation based on attitudes, behaviors, standards, and values. The safety culture encompasses a set of dominant indicators, beliefs, and values about safety that the organization upholds^3^.

Regulatory institutions, universities, and government organizations have come to recognize the critical role of creating and maintaining a safety culture in preventing major accidents^4^. Several studies indicate that for developing a process safety culture, senior managers must be committed to ensuring the safety and wellbeing of their employees, particularly during times of production stress. However, the primary issue is that some obstacles hinder senior managers from demonstrating their values, attitudes, and commitments towards their employees^5^. In order to prevent major accidents, endeavors to enhance workplace safety have transitioned from regulating technical matters and individual mistakes to concentrating on organizational factors. After a series of significant accidents, such as the Chernobyl disaster and the Alpha Piper explosion, studies have demonstrated that inadequate safety culture within these organizations is the primary cause of such major accidents. Multiple research works have indicated that having a strong safety culture has favorable impacts on enhancing safety performance^6^ and diminishing accident rates^7^. Shortly after the Columbia disaster, the Center for Chemical Process Safety (CCPS) of the American Institute of Chemical Engineers (AICHE) discussed the concept of safety culture. The CCPS defined safety culture as a combination of group values and behaviors that determine how process safety is managed. This comprehension has resulted in significant improvements in new research within the field of safety culture.

In recent decades, the safety culture of processes has been identified as a random factor or a contributing factor in many incidents and accidents^8^. The safety culture of processes encompasses hidden and often vague factors such as information issues, noncompliance, failure to detect emerging hazards, role ambiguity, management self-awareness, inadequate communication, and low safety prioritization. These factors can be regarded as covert conditions that contribute to the causation of incidents^9^. If an organization considers safety as an investment rather than just an expense, it can anticipate a decrease in the related losses and costs over time. Moreover, a small investment in short-term safety programs can prevent significant costs that might arise from future disasters. These costs could include compensating workers for damages, lost work time, or legal fees. Furthermore, if the accident is significant, the organization's public credibility will be affected, resulting in financial losses in the market^10^. Many organizations have made attempts to evaluate and enhance their safety culture in order to minimize damages and costs^11^.

Safety issues in process industries have become a significant managerial challenge due to the complexity of processes, chemical substances, advanced equipment, and the interaction between humans and machines^12^. Establishing an active and effective safety culture in these environments is recognized as a fundamental necessity. Therefore, studying and researching in the field of safety culture in process industries, with the aim of improving processes and preventing adverse incidents, is of great importance^13^. Examining various factors that influence safety culture is one of the essential aspects in enhancing the level of safety culture in work environments. These factors encompass a diverse range of social, organizational, and individual elements that play a crucial role in formulating and solidifying an effective safety culture^13^.

Considering the importance of safety culture in process industries and the various factors that can affect this issue, this study was conducted with the aim of determining the effective factors on process safety culture and examining the relationship between them using a combined methods of Hidden content analysis and Fuzzy-Decision making trial and evaluation laboratory (DEMATEL).

The innovation of this study lies in employing a combined approach to identify factors influencing process safety culture and determine their relationships. This approach utilizes specialized knowledge (Delphi technique) and real-world information (literature review) for factor identification. Another feature of this approach is establishing connections between factors based on a fuzzy set decision-making model (Fuzzy-DEMATEL), enhancing result accuracy. While individual techniques of this approach have been used in previous studies, their combination for identifying and establishing relationships between factors influencing process safety culture has not been utilized before.

The objective of this paper is to present an approach for comprehensive identification of factors influencing process safety culture and determining their relationships. The paper is organized as follows: the second section provides a literature review to depict the diversity of factors influencing safety culture. The third section details the study methodology. The fourth section examines the study results. The fifth section discusses the obtained results, and the final section presents conclusions.

## Literature review

Previous studies have identified management/leadership commitment and active employee participation as desirable indicators of safety culture^13–15^. Fernandez et al. conducted a study which identified three crucial components of organizational safety culture: management commitment, employee participation, and safety management system^16^. The results study of Alimohammadi et al. showed that safety culture in the detergent and cleaning industries is comprised of five dimensions: management commitment, training and information exchange, supportive environment, inhibitory factors, and prioritization of safety^17^.

Given the importance of the process safety culture, some researchers have investigated the factors influencing it. Table 1 presents some of these studies.

**Table 1:** The conducted studies to identification factors which influencers process safety culture.

Study	Factors influencing process safety culture	References
Ranking of process safety cultures for risk-based inspections using indicative safety culture assessments	Leadership and commitment, opinion of management about the causes of incident, profit vs safety, safety communication, participation and commitment of employees, contractor management, procedures and rule managemen, incident reporting and analysis, execution and follow-up of audits, personal vs process safety, functioning and roles of supervisors, maintenance management, safety learning, dealing with complexity	^13^
Repeated assessment of process safety in major hazard industries in the Rotterdam region (The Netherlands)	Education and training, launching is safety culture improvement program, realizing concrete safety improvement, strengthening the involvement of personnel, leadership commitment, safety vs productivity, safety communication, participation, vision of senior management on the causes of incident, accident registration and analysis, learning from incident, managing contractor safety, role of supervisors with regard to safety, process safety vs occupational safety, maintenance management (and drift to danger), dealing with procedures, execution and follow-up of audits, complexity/resilience	^18^
The mediating role of safety management practices in process safety culture in the Chinese oil industry	Leadership/management commitment, employee involvement, organizing responsibilities/procedures, safety training, inception and monitoring, communication and coordination	^14^
Assessing process safety culture maturity for specialty gas operations: a case study	Leadership/management commitment, employee involvement	^15^
Nurturing a strong process safety culture	Creating awareness about safety culture topics (Education), Maintaining a sense of vulnerability, Combating normalization of deviance, Performing appropriate and timely hazard/risk assessment, Establishing an independent and unassailable role for safety, Ensuring open and frank communications across all levels	^19^
Process safety culture in the CCPS risk based process safety model	Maintain a dependable practice, develop and implement a sound culture, monitor and guide the culture, establish process safety as a core value provide, strong leadership, establish and enforce high standards of performance, document the process safety culture emphasis and approach, maintain a sense of vulnerability, empower individuals to successfully fulfill their safety responsibilities, defer to expertise, ensure open and effective communications, establish a questioning/learning environment, foster mutual trust, provide timely response to process safety issues and concerns, provide continuous monitoring of performance	^20^
Development of a process safety culture of chemical engineers	Maintaining a sense of vulnerability, establish an imperative for safety, perform valid/timely hazard/risk assessments, ensure open and frank communications, learn and advance the culture	^21^

Based on the studies presented in Table 1, it is evident that multiple factors influence the safety process culture. Different studies have examined various factors and have focused solely on exploring these factors. In this study, an attempt has been made to identify a comprehensive set of influential factors.

## Research method and material

**Figure 1:**
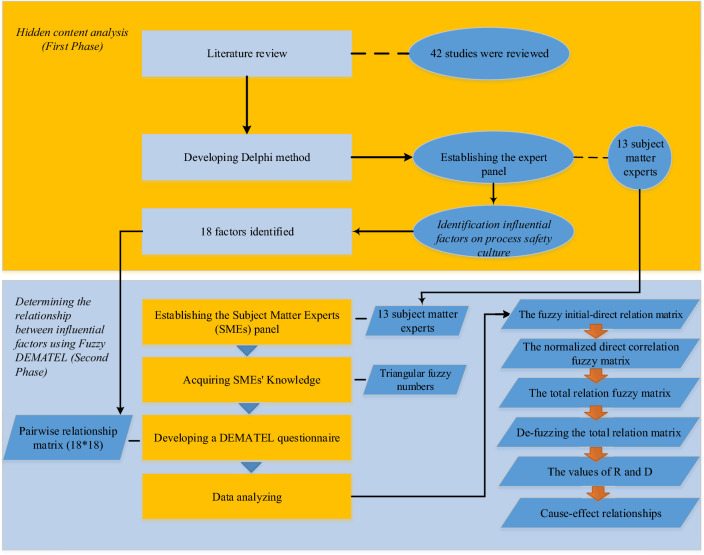
The proposed methodology.

The study consisted of two stages. During the first stage, we identified the factors that have an impact on process safety culture. In the second stage, we used the fuzzy-DEMATEL method to determine the interactions between these factors. Figure 1 depicts the proposed methodology.

### Hidden content analysis (first phase)

In this study, the qualitative approach of hidden content analysis (thematic analysis) was utilized to identify the factors that influence process safety culture. Hidden content analysis is a qualitative research strategy that aims to develop a theory about a phenomenon by identifying its fundamental components and categorizing the relationships between these components within the context and process^22^. Because ensuring safety and preventing accidents necessitate an approach that is applicable in decision-making, this study utilized the hidden content analysis technique in two sequential steps as outlined below.

#### Literature review

**Figure 2:**
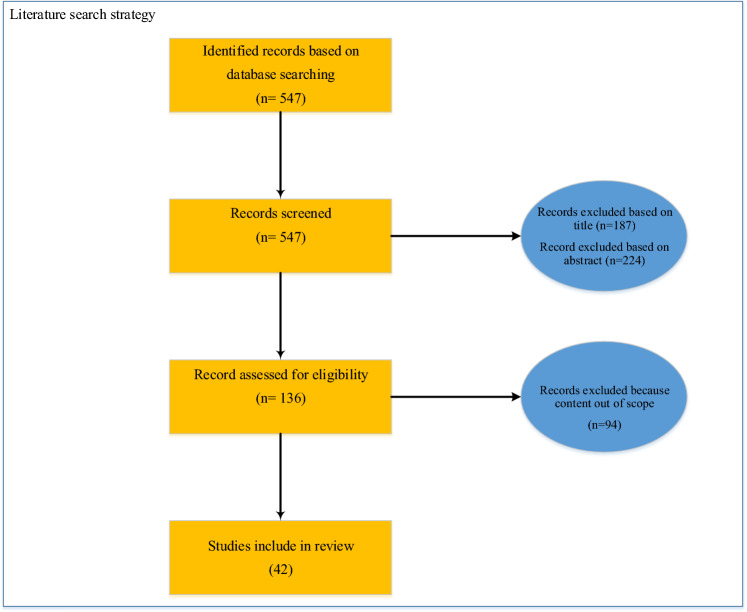
The literature search strategy.

In this step, the factors that have an impact on process safety culture were identified through a literature review. To accomplish this, relevant studies related to process safety culture were searched using key words include as Safety Culture, Process safety culture, and Process safety. The search was done in databases of Web of Sciences, Scopus, PubMed, and Google Scholar from the time between 2000 and 2023. The literature search strategy is illustrated in Fig. 2. In total, 42 studies were reviewed. In total, 42 article were selected to review. To extract factors, an open coding process was utilized. In open coding, factors are named without any constraints^23^.

Given the diversity of factors and for a more comprehensive data analysis, a three-member panel of safety experts was formed. These individuals were university professors with associated degree. The average age and work experience of them were 42 ± 1.38 and 8 ± 0.82, respectively. The selected articles, were reviewed carefully by experts and influential factors were extracted.

#### Developing Delphi method

In order to ensure the accuracy of factors influencing process safety culture and to identify all relevant factors, the Delphi method was used in conjunction with text analysis.

The Delphi method aims to collect and integrate opinions using a series of questionnaires or interviews to establish consensus through participant input. In addition, diverse opinions within a narrower scope are consolidated, as this technique supports continuous refinement. Key features of this method include participant anonymity, iterative and repeated multi-stage processes, controlled feedback, and statistical aggregation. Anonymity is a fundamental aspect of the Delphi method. Participants in a panel do not know each other, and under the influence of group dynamics, participants are not swayed by peer pressures. Therefore, participants are encouraged to express any important views, and this process is repeated until a reliable consensus is reached among the participants. Responses from each round are summarized and reported, allowing participants to reconsider their opinions in light of other participants' responses. Participants are allowed to change their initial responses. Each participant's responses in each round are collected and presented by combining the mean and standard deviation^24^. The Delphi method has been widely used in various fields^24^. Considering the unique characteristics of the Delphi method, this study utilized the technique to identify factors influencing process safety culture. The Delphi method was carried out in five steps as follows:

*Step 1: Establishing the expert panel*

Basically, there is no specific method for determining the number of participants or the preferred panel size for each study. The panel should consist of a group of selected experts without size limitation. However, since there is a need for specialized individuals who have the most knowledge and experience in the relevant field under consideration, the size of the group often remains relatively small^25^. According to Hogarth, the optimal number of members for utilizing the Delphi technique is between six to twelve individuals^26^. However, in typical conditions, a panel usually consists of 10–30 specialists^25^.

In this research, Delphi panel members were selected through purposeful non-probability sampling. Initially, experts and specialists were identified based on their work experience (10–15 years), expertise (academic members with associated professors’ degree), and familiarity with the safety culture and process safety. From this list, 18 individuals were selected. The experts were contacted via phone calls and given a detailed explanation of the study's purpose and methodology. To uphold ethical standards, the participants were guaranteed that any information they provided would remain confidential. If the experts expressed their interest in taking part, they were extended an invitation to join the panel. Ultimately, a total of 13 subject matter experts with diverse backgrounds and expertise participated in the study as members of the expert panel. It should be noted that the appropriate number of panel members is another important consideration in forming the panel. According to the literature of this method, the 13 selected individuals were deemed suitable for the Delphi panel^25,26^.

*Step 2: designing the Delphi Questionnaire*

The Delphi Semi structured questionnaire was designed based on the determined factors. This questionnaire utilized a five-point Likert scale with phrases 'not important,' 'somewhat important,' 'important,' 'very important', and 'extremely important' to measure the factors. These phrases corresponded to scores of 1, 2, 3, 4, and 5, respectively.

*Step 3: determine the number of Delphi rounds*

There are two statistical criteria for deciding whether to continue or stop Delphi rounds. The first criterion is the occurrence of a strong consensus among panel members, determined based on the value of the Kendall's coefficient of concordance (K ˃ 0.5). If such consensus does not occur, the constancy or minimal growth of this coefficient over two consecutive rounds indicates a lack of increase in agreement among members, and the polling process should be discontinued^27^. In This study the number of Delphi round determined using Kendall's coefficient.

*Step 4: Validation and screening of factor*

This process is carried out by comparing the value of the acquisition of each factor with the threshold value $\tilde{S}$. The threshold value is determined through the mental inference of the decision-maker. In this study, the threshold value is considered to be three^28^. If the average score of each factor is less than 3, that index will be excluded.

*Step 5: Identification influential factors on process safety culture*

In the first round, the prepared questionnaire was provided to the experts to score each factor based on a 5-point Likert scale. After experts completed the questionnaires, the average of the factors was calculated.

In the second round of Delphi, factors with an average score of less than three in the first round were eliminated. The confirmed factors from the first round, along with factors extracted by experts, were again presented to the experts through a questionnaire to score each index, similar to the first round. In this round, the average scores from the first Delphi round were also provided for individuals to make decisions based on the overall average. In this round, many experts confirmed their opinions from the first round.

In the three rounds of Delphi, a similar questionnaire from the two round was again provided to the experienced individuals to score each factor, similar to the previous rounds. In this round, as no factors were eliminated or added, the Kendall coefficient was calculated. Since the value of the Kendall coefficient was higher than 0.5, Delphi was stopped at this stage Following the four rounds, a total of 18 factors were determined to be influential on process safety culture.

### Determining the relationship between influential factors using Fuzzy DEMATEL (second phase)

In this phase, the fuzzy-DEMATEL technique was utilized to establish the relationship between the identified factors. The variables considered in this section were the identified influential factors on process safety culture. DEMATEL is a reliable approach for examining relationships among system factors by consolidating group expertise. Its key attribute in the field of multi-criteria decision-making is its capacity to establish relationships and structures among factors^29^. One of its key advantages over other methods such as the Analytical Hierarchy Process (AHP) is that it captures mutual dependencies between system factors through an arrow diagram, which is often overlooked in traditional approaches^30^. To address the uncertainty associated with expert judgment, combining this method with the fuzzy concept yields benefits^31^.

Fuzzy-DEMATEL method is employed to consider multi-criteria interactions and calculate the weights of all criteria^32^. In this study, the Fuzzy-DEMATEL method served three main purposes. Firstly, to calculate the correlation matrix between the influential factors, identify causal factors, and determine the level of influence of each cause and finally establish the cause-effect model regarding the influential factors. The stages involved in performing this task are as follows:

*Step 1: Establishing the Subject Matter Experts (SMEs) panel*

Due to the role of the Delphi panel members in determining the influential factors on the process safety culture, and the good knowledge of them regarding the importance of each factor compared to others, they were invited to SMEs panel. Since all specialists had similar levels of knowledge in the field under investigation, equal weightage was assigned to each of them.

*Step 2: Acquiring SMEs' Knowledge*

Involving SMEs in determining relationship improves the investigation's quality and dependability, resulting in more precise findings. To achieve this, we applied a well-known linguistic approach that utilizes mental categorizations of language variables to tap into the expertise of SMEs. A language variable is a term for a spectrum of values represented by words or sentences in either natural or artificial language. The linguistic scales employed in this technique, along with their associated values, can be found in Table 2. This investigation utilized a range of triangular fuzzy numbers, a method that has been applied in numerous prior studies.

**Table 2:** Linguistic phrases and corresponding fuzzy numbers.

Linguistic terms	Triangular fuzzy number
No effect (No)	(1,1,1)
Very low effect (VL)	(2,3,4)
Low effect (L)	(4,5,6)
High effect (H)	(6,7,8)
Very high effect (VH)	(8,9,9)

*Step 3: Developing a DEMATEL questionnaire*

According to the 18 determined factors, a pairwise comparison matrix of 18*18 has been created. The questionnaires and guidelines for completion were emailed to experts. The experts were requested to express their views on the direct influence of each factor on one another, using the linguistic variables outlined in Table 2. Throughout the two-week data collection period, respondents had convenient access to researchers in case they had any questions about the criteria selection process.

*Step 4: Data analyzing*

Once the experts finished creating the pairwise comparison matrices, the data was extracted and examined. To carry out the fuzzification process, qualitative opinions were initially transformed into fuzzy numbers that can be found in Table 2. Subsequently, the analysis followed the steps provided below.

*Step 1:* The fuzzy initial-direct relation matrix (${\tilde{\text{E}}}$) is generated through the gathering of expert opinions from those involved in the study. The opinion matrices for each variable provided by the experts are averaged to create ${\tilde{\text{E}}}$. Equation (1), where 'p' indicates the number of experts participating in the study, is utilized to perform this procedure.

1 $$\widetilde{E}=\frac{{\widetilde{E}}^{\langle 1\rangle }+{\widetilde{E}}^{\langle 2\rangle }+\dots +{\widetilde{E}}^{\langle p\rangle }}{p}$$

$$\widetilde{E}=\left[\begin{array}{ccc}0& \dots & {\widetilde{E}}_{1n}\\ \vdots & \ddots & \vdots \\ {\widetilde{E}}_{n1}& \dots & 0\end{array}\right], {\widetilde{e}}_{ij}=({l}_{ij},{m}_{ij},{u}_{ij})$$

*Step 2:* In the second stage, the normalized direct correlation fuzzy matrix (F ~) was derived. The process involved utilizing Eqs. (2) and (3).

2 $$\widetilde{F}=\frac{\widetilde{E}}{\gamma }$$

3 $$\gamma =max\sum_{j=1}^{n}{u}_{j}$$

$$\widetilde{F}=\left[\begin{array}{ccc}{\widetilde{F}}_{11}& \dots & {\widetilde{F}}_{1n}\\ \vdots & \ddots & \vdots \\ {\widetilde{F}}_{n1}& \dots & {\widetilde{F}}_{nn}\end{array}\right], {\widetilde{f}}_{ij}=\frac{{\widetilde{e}}_{ij}}{\gamma }=(\frac{{l}_{ij}}{\gamma },\frac{{m}_{ij}}{\gamma },\frac{{u}_{ij}}{\gamma })$$

*Step 3:* The total fuzzy matrix (T ~) was derived by normalizing the direct correlation fuzzy matrix and applying Eqs. (4)–(6).

4 $$Matrix\left[{l}_{ij}^{\mathrm{^{\prime}}}\right]= {F}_{l}\times {\left(1-{F}_{l}\right)}^{-1}$$

5 $$Matrix\left[{m}_{ij}^{\mathrm{^{\prime}}}\right]= {F}_{m}\times {\left(1-{F}_{m}\right)}^{-1}$$

6 $$Matrix\left[{u}_{ij}^{\mathrm{^{\prime}}}\right]= {F}_{u}\times {\left(1-{F}_{u}\right)}^{-1}$$

*Step 4:* De-fuzzing the total relation matrix using Eq. (7).

7 $${t}_{ij}=\frac{1}{4}\left({l}_{ij}^{\mathrm{^{\prime}}}+2{m}_{ij}^{\mathrm{^{\prime}}}+{u}_{ij}^{\mathrm{^{\prime}}}\right)$$

$$T=\left[\begin{array}{ccc}{t}_{11}& \dots & {t}_{1n}\\ \vdots & \ddots & \vdots \\ {t}_{n1}& \dots & {t}_{nn}\end{array}\right]$$

*Step 5:* The calculations for the values of R and D for each variable are derived from the de-fuzzed matrix components of the overall relationship, utilizing Eqs. (8) and (9). The value of D indicates the degree of influence of each variable on other variables. Also, R indicates the influence of each variable on other variables.

8 $$D=\sum_{j=1}^{n}{t}_{ij}, \left(j=\mathrm{1,2},3\dots ,n\right)$$

9 $$R=\sum_{i=1}^{n}{t}_{ij}, \left(i=\mathrm{1,2},3\dots ,n\right)$$

*Step 6:* Establishing causal relationships. During this stage, the indexes of D + R and D − R were calculated. The D + R index signifies the extent of interaction between each factor with other factors, whereas the D − R index reveals the nature of the interaction. A higher D + R value indicates a stronger level of interaction with other factors. Furthermore, if the D − R value is positive, the considered factor plays a causal role, but if it is negative, the factor is seen as an effect^33^.

## Results

### Factors affecting the process safety culture

This study was conducted with the aim of determining the factors influencing the process safety culture and their relationship. The method of hidden content analysis was used to identify the influential factors. In this method, the factors were identified through two stages of literature review and Delphi method. Based on the literature review, 29 influential factors were identified (Table 3).

**Table 3:** The influential factors of process safety culture based on the literature review.

Row	Influential factors
1	Leadership and commitment
2	Opinion of management about the causes of incident
3	Profit vs safety
4	Safety communication
5	Participation and commitment of employees
6	Contractor management
7	Procedures and rule management
8	Incident reporting and analysis
9	Execution and follow-up of audits
10	Personal vs process safety
11	Functioning and roles of supervisors
12	Maintenance management
13	Safety learning
14	Dealing with complexity
15	Education and training
16	Launching is safety culture improvement program
17	Realizing concrete safety improvement
18	Safety vs productivity
19	Role of supervisors with regard to safety
20	Process safety vs occupational safety
21	Complexity/resilience
22	Organizing responsibilities
23	Inception and monitoring
24	Risk assessment in different ways
25	Creating awareness about safety culture topics (Education)
26	Maintaining a sense of vulnerability
27	Combating normalization of deviance
28	Establishing an independent and unassailable role for safety
29	Asses the process safety culture

Based on the proposed methodology, in the subsequent step, the extracted items were presented to the Delphi panel. Initially, the identified factors were provided to the experts to express their opinions and articulate any similar aspects about the factors. Experts were also asked to indicate if they had any factors in mind other than the ones mentioned. Based on expert opinions, the factors underwent a reviewed. As a result of this stage, 21 factors determined to design Delphi questionnaire. The reason for reducing the number of factors during this stage was the merging of some factors with one another due to their similarity in name or function. The panel of experts selected for this stage of the research comprised 13 academic individuals (Associated professors) who specialize in process safety. The experts' average age was 45.82 ± 2.14, and their average work experience was 12.57 ± 0.86 years. The Delphi study was applied three rounds. Table 4 displays the results of Delphi technique.

**Table 4:** The influential factors of process safety culture based on the Delphi technique.

Row	First Delphi stage	Mean	Second stage Delphi	Mean	Third stage Delphi	Mean
1	Commitment in organization management and leadership	4.25	Organization management's commitment to safety	4.43	Organization management's commitment to safety	4.43
2	Safety against production	3.21	Safety in production	2.87	Open and frank safety communication	3.92
3	Open and safe communication	3.84	Open and frank safety communication	3.92	Employee participation and commitment	4.23
4	Employee participation and commitment	4.12	Employee participation and commitment	4.23	Contractor management	3.97
5	Contractor management	3.97	Contractor management	3.97	Safety policies and regulations	4.17
6	Policies and safety regulations	4.21	Safety policies and regulations	4.17	Incident reporting system	3.14
7	Event reporting system	3.14	Incident reporting system	3.14	Analysis and learning from incident	3.68
8	Access to safety information	3.56	Incident analysis	2.84	Access to process information	3.56
9	Monitoring/inspection	3.94	Learning from incidents	3.48	Monitoring/inspection	3.94
10	Maintenance management	4.06	Access to safety information	3.56	Maintenance management	4.06
11	Training and education	4.15	Monitoring/inspection	3.94	Education and Training	4.2
12	Simplification or avoidance of complexity	3.58	Maintenance management	4.06	Simplification or avoidance of complexity	3.58
13	Continuous improvement program in safety culture	2.78	Safety training	4.2	Process safety vs. personal safety	3.74
14	Execution and follow-up of audits	2.46	Simplification or avoidance of complexity	3.58	Risk assessment and management	3.82
15	Organizing responsibilities	2.21	Process safety vs. personal safety	3.74	Incentive and punishment system in safety field	4.16
16	Process safety vs individual safety	3.74	Risk assessment and management	3.82	Safety permit system	3.69
17	Risk assessment and management	3.82	Incentive and punishment system in safety field	4.16	Perceived organizational support for safety	3.84
18	Incentive and punishment system in safety area	4.16	Safety permit system	3.69	Change management	3.74
19	Safety permit system	3.69	Perceived organizational support for safety	3.84	Reliability = 0.825 Kendall coefficient = 0.625	
20	Perceived organizational support for safety	3.84	Change management	3.74		
21	Change management	3.74	Reliability = 0.825			

The Delphi technique finally identified 18 influential factors on process safety culture.

### The relationships between identified influential factors

In the first phase 18 factors identifaed as influential factors on process safety culture. However, during the initial stage, the outcomes were qualitative, and it was unclear how each factor affected the process safety cultures. Additionally, the relationship between these factors remained uncertain. To overcome this limitation, the fuzzy-DEMATEL method was employed to quantify the results. DEMATEL is a powerful technique for analyzing the connections among system factors by utilizing collective knowledge. The expert panel for this phase of the study resembled the Delphi panel used in the first phase. Since the group was homogeneous, there was no need to assign different weights to the experts. By utilizing their expertise and the linguistic variables provided in Table 2, the experts indicated the direct impact of each factor on one another using the generated DEMATEL questionnaire. Given the vast number of factors involved, the identified factors were coded to facilitate their usage. Table 5 depicts the results of this coding process. Subsequently, the linguistic estimates were converted into fuzzy numbers, resulting in a matrix of direct relationships known as Table 6. This matrix demonstrates the direct influence of factor i on factor j. When performing the calculations, the diagonal elements of the matrix were set to zero if i = j^34^.

**Table 5:** Results of coding the factors influencing process safety culture.

Factors	Code
Organization management's commitment to safety	A1
Open and frank safety communication	A2
Employee participation and commitment	A3
Contractor management	A4
Safety policies and regulations	A5
Incidents reporting system	A6
Analysis and learning from incidents	A7
Access to process information	A8
Monitoring/inspection	A9
Maintenance management	A10
Education and training	A11
Simplification or avoidance of complexity	A12
Process safety vs. personal safety	A13
Risk assessment and management	A14
Incentive and punishment system in safety field	A15
Safety permit system	A16
Perceived organizational support for safety	A17
Change management	A18

**Table 6:** Initial direct correlation matrix (mean matrix).

	A1	A2	A3	…	A16	A17	A18
A1	(0,0,0)	(5.85,6.85,7.77)	(7.08,8.08,8.54)	…	(5.85,6.85,7.38)	(6.92,7.92,8.38)	(5.85,6.85,7.38)
A2	(3.77,4.54,5.23)	(0,0,0)	(5.69,6.69,7.31)	…	(3.92,4.85,5.46)	(3.62,4.54,5.46)	(3.92,4.85,5.77)
A3	(4.62,5.46,5.92)	(5.46,6.38,7)	(0,0,0)	…	(5.15,6.08,6.69)	(3.77,4.69,5.62)	(5.46,6.38,7)
…	…	…	…	…	…	…	…
A16	(2.69,3.46,4.15)	(3,3.62,4.23)	(4.69,4.46,5.92)	…	(0,0,0)	(3.77,4.54,5.23)	(4.46,5.15,5.54)
A17	(3.54,4,38,5.15)	(4.62,5.46,6.31)	(5.62,6.54,7)	…	(4,4.69,5.31)	(0,0,0)	(2.54,3.31,4.08)
A18	(2.62,3.31,3.92)	(5.85,6.85,7.77)	(3.92,4.85,5.77)	…	(3.23,3.92,4.62)	(2.38,3.15,3.92)	(0,0,0)

After obtaining the fuzzy direct relationship matrix, the normalized fuzzy direct relationship matrix and the total fuzzy relationship matrix were calculated and prepared. In the next step, to convert the total fuzzy relationship matrix into a comparable value, defuzzification was done on the total relationship matrix (Table 7). The average value of the de-fuzzified total relationship matrix (0.113) was used as the threshold. Therefore, an impact score of one factor on another factor ≥ 0.113 indicates a significant effect of the causal factor on the effect factor.

**Table 7:** De-fuzzified total relation matrix.

	A1	A2	A3	A4	A5	A6	A7	A8	A9	A10	A11	A12	A13	A14	A15	A16	A17	A18
A1	0.09	0.15	0.17	0.16	0.16	0.17	0.16	0.15	0.17	0.16	0.17	0.12	0.14	0.17	0.16	0.17	0.16	0.15
A2	0.11	0.07	0.14	0.12	0.12	0.14	0.13	0.12	0.12	0.12	0.13	0.09	0.11	0.13	0.12	0.13	0.11	0.12
A3	0.12	0.13	0.10	0.13	0.14	0.15	0.14	0.13	0.14	0.13	0.15	0.11	0.12	0.15	0.13	0.14	0.12	0.13
A4	0.08	0.08	0.11	0.07	0.12	0.11	0.10	0.08	0.12	0.11	0.11	0.08	0.09	0.12	0.11	0.11	0.10	0.10
A5	0.13	0.11	0.15	0.14	0.10	0.16	0.14	0.13	0.15	0.15	0.14	0.12	0.12	0.15	0.13	0.16	0.13	0.14
A6	0.10	0.10	0.12	0.11	0.10	0.08	0.13	0.10	0.13	0.12	0.11	0.09	0.10	0.13	0.11	0.12	0.09	0.10
A7	0.11	0.10	0.11	0.11	0.12	0.14	0.08	0.10	0.12	0.12	0.13	0.10	0.10	0.13	0.10	0.13	0.09	0.11
A8	0.10	0.10	0.11	0.10	0.13	0.12	0.12	0.07	0.13	0.13	0.13	0.11	0.11	0.14	0.10	0.13	0.09	0.12
A9	0.11	0.11	0.12	0.13	0.13	0.13	0.12	0.12	0.09	0.14	0.13	0.10	0.11	0.14	0.12	0.14	0.11	0.12
A10	0.08	0.07	0.10	0.09	0.10	0.09	0.09	0.09	0.11	0.07	0.10	0.09	0.09	0.10	0.08	0.11	0.09	0.08
A11	0.12	0.13	0.15	0.14	0.14	0.15	0.14	0.13	0.13	0.13	0.10	0.11	0.12	0.15	0.12	0.14	0.13	0.13
A12	0.06	0.06	0.09	0.08	0.09	0.09	0.08	0.08	0.09	0.10	0.08	0.05	0.09	0.11	0.08	0.09	0.07	0.09
A13	0.09	0.09	0.10	0.09	0.11	0.10	0.11	0.10	0.11	0.10	0.10	0.09	0.06	0.11	0.08	0.11	0.09	0.11
A14	0.13	0.12	0.15	0.14	0.16	0.16	0.15	0.13	0.16	0.14	0.15	0.13	0.13	0.11	0.13	0.16	0.14	0.13
A15	0.09	0.10	0.12	0.11	0.10	0.12	0.11	0.08	0.10	0.10	0.11	0.08	0.08	0.11	0.06	0.11	0.11	0.09
A16	0.09	0.09	0.11	0.11	0.11	0.10	0.10	0.09	0.11	0.11	0.11	0.08	0.09	0.11	0.10	0.08	0.10	0.10
A17	0.10	0.11	0.13	0.10	0.11	0.12	0.11	0.10	0.12	0.10	0.12	0.09	0.09	0.12	0.11	0.12	0.07	0.10
A18	0.09	0.09	0.11	0.11	0.11	0.11	0.11	0.09	0.11	0.11	0.11	0.10	0.10	0.12	0.09	0.11	0.09	0.07

In the next step, the D and R values were calculated to determine the cause-and-effect variables. The sum of the values of each row in the De-fuzzified matrix of the total relationship (D) indicates the degree of influence of each variable on other variables (Table 8), while the sum of the values of each column (R) indicates the influence of each variable on other variables^35^.

**Table 8:** The values of D, R, D + R, and D − R.

Factor name	Code	D	R	D + R	D − R	The role of factor
Organization management's commitment to safety	A1	**2.78**	1.79	4.57	**0.99**	Cause
Open and frank safety communication	A2	2.14	1.80	3.94	0.34	Cause
Employee participation and commitment	A3	2.34	2.21	4.55	0.13	Cause
Contractor management	A4	1.81	2.04	3.85	− 0.23	Effect
Safety policies and regulations	A5	2.47	2.13	4.60	0.34	Cause
Incidents reporting system	A6	1.96	2.24	4.20	− 0.28	Effect
Analysis and learning from incidents	A7	2.02	2.13	4.15	− 0.11	Effect
Access to process information	A8	2.04	1.89	3.93	0.15	Cause
Monitoring/inspection	A9	2.15	2.19	4.34	− 0.04	Effect
Maintenance management	A10	1.63	2.16	3.79	**− 0.53**	Effect
Education and training	A11	2.36	2.17	4.53	0.19	Cause
Simplification or avoidance of complexity	A12	**1.48**	**1.72**	**3.20**	− 0.24	Effect
Process safety vs. personal safety	A13	1.75	1.85	3.60	− 0.10	Effect
Risk assessment and management	A14	2.52	**2.31**	**4.83**	0.21	Cause
Incentive and punishment system in safety field	A15	1.76	1.93	3.69	− 0.17	Effect
Safety permit system	A16	1.77	2.25	4.02	− 0.48	Effect
Perceived organizational support for safety	A17	1.89	1.86	3.75	0.03	Cause
Change management	A18	1.82	1.98	3.80	− 0.16	Effect

Significant values are in bold.

The values of D + R and D − R are another important part the results obtained from the fuzzy DEMATEL method. The value of D + R indicates the degree of interaction between the desired variable and other variables, while the value of D − R indicates the type of interaction of each variable with other variables. A higher value of D + R suggests a greater level of interaction that the variable has with other variables. Additionally, if the value of D − R positive, it means that the desired variable plays a causal role, whereas a negative value D − R indicates that the desired variable plays an effect role^35^. The D + R and D − R values can be found in Table 8.

**Figure 3:**
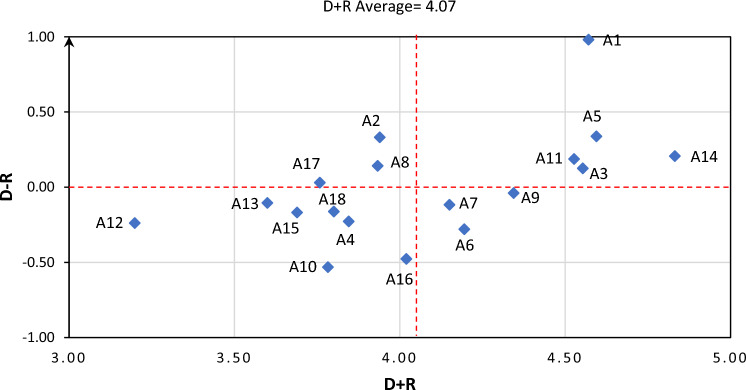
INRM of factors, the relationship between factors.

Finally, the influential network relations map (INRM) of factors was created (Fig. 3). In this diagram, D + R and D − R represent the level of interaction and influence, respectively.

Based on the results (Fig. 3), eight variables were identified as causes, while ten variables were classified as effects. In the context of the fuzzy DEMATEL technique, causes refer to variables that significantly impact the system. In the context of process safety culture, causes can be understood as variables that directly contribute to creating the safety culture. As evident from Fig. 3, the most influential factor is related to the commitment of organization managements to safety.

## Discussion

### Factors affecting the process safety culture

The first phase of study identified 18 influential factors on process safety culture. In the following, these factors have been discussed separately.

#### Organization management's commitment to safety

In various industries and organizations, there has been discussion about the importance of researching management's commitment to safety as a crucial factor in establishing a culture of safety. The level of importance and priority that managers place on occupational safety plays a significant role in promoting a positive safety culture^36^. One of the most important steps that can be taken to establish a culture of safety is clear communication with employees about safety priorities. This involves informing them of the high priority given to safety, emphasizing that it is regarded as the top business issue. The level of priority assigned to occupational safety within a business is directly linked to the management's commitment to health and safety concerns^37^. Such a commitment can take many forms, including identifying and evaluating potential risks, complying with health and safety laws and regulations, providing employee training, establishing appropriate facilities and technical equipment, promoting a culture of safety, and fostering a sense of motivation to comply with safety protocols. Research has indicated that the establishment of a safety culture within an organization is directly linked to management's commitment to safety.

#### Open and frank safety communication

Effective communication is essential within industrial organizations to promote a culture of safety. When employees are encouraged to speak openly and honestly, they can identify potential hazards and suggest ways to improve safety. This type of communication also allows employees to share information and encourage safe habits among their colleagues. Establishing open lines of communication between managers and employees can significantly improve a company's approach to process safety. By fostering an environment where employees feel comfortable expressing their concerns and collaborating with management, organizations can make better decisions and implement more effective safety measures. Ultimately, creating a culture of open and frank communication can contribute to a reduction in workplace injuries and accidents. Effective safety communication is crucial for reducing employee accidents in the workplace. It goes beyond simply sharing information about workplace safety, as it also has the power to influence employees' behavior and attitudes towards safety^38^. Effective safety communication has been shown to have a positive impact on safety performance. Insufficient or inappropriate safety interaction between employees and senior management may be due to a lack of emphasis on constructive communication and feedback about workplace safety. One reason why safety communication might be weak is the absence of a positive safety culture within an organization^39^. A study conducted by Parker and his colleagues found that establishing open communication channels between managers and employees can result in significant improvements to process safety culture. The research showed that such communication can lead to higher job satisfaction among employees, increased organizational commitment, and improved safety performance^40^.

#### Employee participation and commitment

The influence of employee participation and commitment on process safety culture is a critical issue in safety management. Safety literature has demonstrated that with the combined efforts of managers and employees, a positive safety culture can be established. Therefore, one-sided actions taken by managers (e.g., providing and implementing safe work laws, procedures, plans, and policies) to ensure healthy and safe working conditions are not sufficient. It is also crucial for employees to comply with these regulations and guidelines and actively participate in safety-related matters. Employee participation refers to actions that may not directly contribute to an individual's safety, but instead help create an environment that supports occupational safety. Safety participation refers to voluntary actions that go beyond the scope of employees' safety-related responsibilities^41^. Extraordinary behaviors encompass actions such as supporting fellow workers, voluntarily participating in safe practices, attending safety meetings, promoting safety programs in the workplace, taking initiative to improve safety, and enhancing overall safety in the workplace.

#### Contractor management

The influence of contractor management on process safety culture is a critical issue in health and safety within the industry. Research has shown that increasing transparency in contractor management can lead to significant improvements in process safety culture. Risk analysis, proper planning, and providing information to contractors can increase awareness and emphasis placed on safety issues, resulting in an improvement in process safety culture. Additionally, involving contractors in decision-making and project implementation processes can help highlight the importance of safety issues and enhance process safety culture. Choosing contractors with adequate expertise and experience in the relevant areas of a project can enhance safety and promote a culture of safety. Experienced contractors in the field of health and safety can further support a culture of safety in the workplace. Moreover, enhancing the training and awareness of contractors can significantly improve process safety culture. Raising contractors' awareness about safety hazards can result in increased safe behaviors and improvements in safety culture.

#### Safety policies and regulations

Safety policies and regulations are a significant factor in shaping the safety culture of processes. Given the technical complexity of chemical industries, it is essential to have operational methods for managing work. A study conducted on Norway's oil and gas industry revealed that safety regulations and policies constitute the primary components of their safety management approach^42^. Incorporating safety measures and adhering to safety policies in refining industries can enhance the safety culture of processes.

#### Incidents reporting system

The incident reporting system is another factor that impacts safety culture. It is a vital tool to enhance process safety culture in industries. This system consists of procedures and processes used to gather, analyze, and report events and errors related to the process. It identifies weaknesses and offers suggestions for enhancing the process safety system.

#### Analysis and learning from incidents

Analysis and learning from incidents are a crucial tool in improving the safety culture within process industries. Through this tool, adverse events and incidents are systematically examined to identify the contributing factors that led to their occurrence. This enables prevention of similar incidents in the future. The impact of analysis and learning from incidents on process safety culture can be classified into two categories—positive and negative developments. In the positive category, a thorough analysis of contributing factors can help prevent similar incidents in the future, leading to improved process safety. In addition, incident analysis can be utilized as an educational tool to enhance the safety culture in process industries. However, improper use of incident analysis and lessons learned may have negative consequences such as rendering the activity ineffective or leading to further mistakes and errors. Therefore, to improve the process safety culture in these industries, it is crucial to use incident analysis appropriately and share the results with employees. Furthermore, it is essential to improve the organizational culture, and increase knowledge and awareness among employees in the field of process safety to ensure sustained effectiveness of this tool.

#### Access to process information

Having access to process and safety information is another factor that can contribute to improving the safety culture in process industries. Accurate and comprehensive information about industrial processes and associated risks enhances employee understanding of incident causes and enables them to adopt appropriate safety measures during their work processes. Furthermore, access to process information empowers managers to identify process strengths and weaknesses, and take necessary preventive measures to improve process safety^43^. Furthermore, having access to process and safety information can be an effective educational tool for promoting process safety culture. This approach helps employees become familiar with potential hazards in their work processes and understand appropriate preventive measures for avoiding accidents^44^.

#### Monitoring/inspection

Monitoring and inspection have been recognized as two key factors in enhancing process safety culture. These activities can be conducted both internally and externally, with internal monitoring and inspection being carried out by employees and managers within the organization, while external monitoring and inspection are performed by government and independent organizations outside of the company. By conducting monitoring and inspection, process strengths and weaknesses can be identified, leading to improved process safety. Additionally, these activities can foster trust and confidence among employees in regards to process safety, leading to increased accuracy in performing tasks. Studies have demonstrated that performing monitoring and inspection activities can be an effective solution for improving process safety culture in process industries.

#### Maintenance management

One of the factors that had a significant impact on the safety culture of the process examined in this study was maintenance management. This factor encompassed activities such as planning, executing, controlling, and maintaining and repairing machinery, equipment, and systems. In general, carrying out periodic and preventive maintenance on machinery and equipment can reduce the likelihood of potential hazards and accidents occurring during the production process, thereby enhancing reliability and safety culture.

#### Education and training

Education and training are considered fundamental in improving process safety culture, and have received significant attention from industrial researchers. Regular and structured education and training programs foster employee commitment to safety culture and reduce the likelihood of industrial accidents. Studies have shown that creating a safety culture requires adequate education, and organizations can only achieve their goals by encouraging individuals to acquire practical knowledge and skills. Therefore, it is necessary for education to be continuous to establish a safety culture^45^.

#### Simplification or avoidance of complexity

Simplification is one of the factors that plays a role in the safety culture of processes. The utilization of sophisticated equipment and complex control systems can contribute to an increase in human error, risks, and accidents. As a result, streamlining processes and avoiding complexity can promote the enhancement and advancement of occupational process safety. Simplification is considered one of the fundamental principles of inherent safety. The implementation of a simplification strategy for equipment and procedures can enhance safety by minimizing operator error^46^. This approach has the potential to increase work morale, job satisfaction, trust in the organization, and ultimately foster a positive attitude towards safety concerns. Furthermore, it can facilitate the ease of working with equipment and procedures, thereby promoting the betterment of safety culture.

#### Process safety vs. personal safety

Process safety versus personal safety was another important factor in shaping process safety culture. Process safety encompasses all methods, standards, and processes used to ensure safety at the level of industrial systems, whereas personal safety refers to the set of activities carried out by individuals to perform their work processes safely. As process safety emphasizes controlling potential hazards in industrial systems and maintaining safety, it can significantly contribute to reducing the number of safety incidents within an organization^47^. Several studies have demonstrated that a robust safety system at the organizational level can enhance overall safety and promote a positive attitude towards safety among employees. As a result, process safety is deemed to be one of the factors contributing to the development of process safety culture^48^.

#### Risk assessment and management

Risk assessment and management is another factor involved in the safety culture of a process. One of the significant effects of risk assessment on the safety culture of a process is the increased awareness of employees about the hazards present in the work environment. Risk assessment can identify various hazards in industrial processes, and the information obtained from this process can serve as a strong foundation for designing and implementing safety processes at the organizational level. As such, risk assessment can act as a catalyst to strengthen the safety culture at the organizational level. Considering the significant role of risk assessment in process safety, it has been regarded as the cornerstone of process safety management. Risk assessment enhances employees' awareness and knowledge of hazards, and provides them with the necessary means to deal with them effectively. Consequently, this can lead to improving employees' safety behaviors, promoting positive attitudes towards safety measures, increasing their participation in safety-related activities, and strengthening the safety culture at the organizational level.

#### Incentive and punishment system in safety field

The incentive and punishment system is an effective approach for enhancing safety culture in industrial settings. By reinforcing the importance of safety and accident prevention, the incentive and punishment system boosts employees' awareness of these critical issues. Through implementation of a robust incentive and discipline system, employees recognize that safe behaviors are rewarded while violation of these behaviors results in punishment. This increased awareness leads to a positive attitude towards safety, promotes greater employee participation in safety initiatives, and ultimately enhances safety culture. Because the design and implementation of an effective incentive and punishment system to improve safety culture in process industries depends on the specific conditions and work environment of each organization, it should not be viewed as a universal solution. Nevertheless, studies indicate that having an incentive and punishment system in place within an organization can lead to improved safety culture^49^.

#### Safety permit system

The safety permit system is a widely used safety policy in many process industries to prevent accidents and occupational hazards. Under this system, a safety permit must be obtained before carrying out hazardous processes to confirm that all the necessary safety conditions for the process, such as using safety equipment and protective coverings, have been provided. By reducing accidents, this system enhances employees' positive attitude towards safety culture. Moreover, the presence of such systems raises the importance of safety from employees' perspective and helps improve safety culture.

#### Perceived organizational support for safety

The findings of this study indicate that perceived organizational support is one of the influential factors in shaping safety culture. Perceived organizational support refers to the degree to which employees believe that their participation, health, and well-being are valued by the organization^50^. When employees sense that the organization cares about them and provides them with adequate safety equipment, they are more likely to comply with safe and low-risk behavior. Perceived organizational support has a direct and indirect impact on safety outcomes by bolstering organizational identification. An organization that regards the preservation of safety and health as a moral value effectively communicates this value to its employees, thereby reducing the likelihood of their deviation from established safety standards^51^.

#### Change management

The change management process is another influential factor in shaping safety culture. By change management, we refer to the consideration of safety issues in implementing changes to industrial processes. Risk assessments should be conducted before and after changes to identify and control hazards, and employees should receive necessary training on the changes. Change management can enhance safety culture by increasing transparency and communication within the organization. By providing transparent and honest information to employees about the changes made and receiving feedback from them, the level of trust and satisfaction of employees with the organization can be increased, leading to improvement in safety culture. In addition to building trust, providing training and raising awareness among employees on safety issues is also crucial in the change management system. By offering appropriate training and guidance to employees on safety, change management can enhance the organization's safety capabilities and strengthen its safety culture. Furthermore, having such systems in place can increase the perceived importance of safety within the organization from the employees' perspective, leading to improved attitudes towards safety and an overall increase in safety culture^52^.

### The relationships between identified influential factors

In the second phase, the relationship between identified factors examined using Fuzzy-DEMATEL technique. Based on the results of this phase, eight variables were identified as causes, while ten variables were classified as effects. Results revaluated that organization management's commitment to safety had the highest weight among factors.

Management's commitment to safety is a key player in creating and maintaining a safety culture in any organization. When managers explicitly commit to the safety of employees and the work environment, it influences all members of the organization. A culture of safety is developed by implementing specific standards, procedures, and processes that are designed to ensure safety by managers. In general, it can be stated that the implementation of all identified influential factors depends on the commitment of organizational management. Therefore, this factor is considered a key player and one of the most influential factors.

Open communication is another factor that has been identified as a cause. One of the most important methods for engaging employees in creating a safety culture is to create an open environment for providing safety opinions and suggestions. Employees should feel confident that their opinions and ideas for safety improvements are valued and taken seriously by the organization. Furthermore, for the implementation of factors such as access to information, Incentive and punishment system, training, incident reporting systems, risk assessment, contractor management, permit systems, etc., open communication between different levels of the organization is necessary. Therefore, this factor is essential for ensuring the implementation of other factors and is considered a cause.

Employee participation is a fundamental factor in creating and strengthening a safety culture in organizations. When employees are actively involved in safety processes and contribute their perspectives, they will collaborate as a cohesive team to implement identified influential factors. Without employee participation, the implementation of factors that create a safety culture will remain enveloped in ambiguity. Therefore, this factor is also considered a cause.

Safety policies and regulations play a crucial role in creating and maintaining a culture of safety in organizations. These laws and standards consist of guidelines and requirements that are designed to preserve and enhance the safety of employees and the work environment. They also play a role in determining and validating safety processes. They encompass various stages, ranging from hazard identification and risk assessment, safety measures, workplace and facility design, selection and use of safety equipment, to safety training for employees and reporting accidents and incidents, outlining necessary processes and requirements. Considering the direct impact of this factor on the creation of other factors and, subsequently, the establishment of a safety culture, this factor is also considered a cause.

Another factor that is considered a cause is the factor of access to process information. For the implementation of certain identified factors, such as risk assessment and management, training, monitoring and inspection, etc., access to process safety information is necessary. Transparency is a key aspect in management, which plays a vital role in improving employee participation. How can we expect employees to participate in the implementation of process safety measures if they do not have information about them? Therefore, access to process information is considered a key factor.

The implementation of many identified factors, such as permit systems, work procedures, incident reporting, change management, etc., requires training. Therefore, this factor is also considered a cause. Another factor that has been identified as a cause is perceived organizational support. This type of support refers to the messages, behaviors, and measures provided by managers and top levels of the organization, demonstrating that safety culture and well-being are valued and prioritized. Since employees play a primary and key role in creating a safety culture, positive perception in this area from the organization can have a significant impact on strengthening a positive attitude towards safety among employees.

In the context of the fuzzy DEMATEL technique, effects refer to variables that are influenced by the system. Regarding process safety, effects can be seen as variables that contribute to the creating of culture. The factors identified as effects are influenced by the causal factors, and it can be said that they play an indirect role in creating a safety culture.

Improving safety culture can be achieved by prioritizing cause factors, as suggested by the study results. Nevertheless, it is important to remember that all the mentioned factors are significant and should not be disregarded.

The results reveals that the Organization management's commitment to safety factor had the greatest influence among all of the factors. Management's commitment to safety can serve as a behavioral model for employees within the organization. When employees perceive that their organization values safety, they are more likely to strengthen their own personal beliefs about safety and be more cautious in their safety-related behaviors.

The social exchange theory is used to explain how management behavior shapes employee perceptions and influences employee behavior^53^. According to the social exchange theory, voluntary behavior is prompted by the norm of reciprocity, meaning that individuals learn about social norms regarding their obligations as much as they fulfill mutual behaviors in official commitments. When individuals fulfill their social commitments, the process of exchange takes place. In terms of safety, when supervisors and managers convey their interest in safety to employees by valuing safety improvement, employees believe that the organization has a positive orientation towards safety, which in turn increases the likelihood of stimulating or exchanging ideas among employees regarding safety issues^54^ and participation in other safety-related activities^55^.

Management's commitment to safety has a significant impact on the prioritization and decision-making processes related to resource consumption, business process design, and safety standards. As a result, since management's commitment to safety affects employees' positive attitudes toward safety and the provision of resources and processes related to safety, it is clear that this factor has the greatest influence on other factors. In other words, without management's commitment to safety, it cannot be expected that other factors effective in creating a safety culture within the organization will emerge, because these factors are reliant on management's attitude and decision-making regarding safety. A safety culture can only be effectively developed when leaders and employees actively participate in organizational safety management. If there are no essential management methods such as goals, policies, initiatives, organizational structure, and resource allocation, the development of a safety culture cannot be achieved at its fullest potential^56^. Safety management practices refer to the policies, strategies, procedures, and activities that organizations implement to ensure the safety of their employees. These practices are essential components of an effective organizational safety management system. Safety management systems and practices are precursors to the development of a process safety culture. Wiegmann and his colleagues carried out research in the aviation industry, and their findings indicated that organizational commitment was one of the most robust elements of safety culture, while reward systems were among the weakest aspects of the studied organization's safety culture^57^. Based on the obtained results, the safety culture can be enhanced by improving the organizational management's attitude towards safety. To improve the management's attitude, it is recommended to conduct training courses and provide economic justification for investment in the safety domain.

The study findings revealed that the most significant interaction was associated with the risk assessment and management aspect. The process of risk assessment and management is a crucial element in maintaining a safety culture, which involves the review and analysis of existing hazards, their severity assessment, and providing safety solutions to mitigate such risks. Given its importance, this process must be conducted with utmost care and coordination. In order to execute the risk assessment and management process effectively, there is a requirement for adequate information on processes, materials, and procedures. For this reason, communication with other safety factors like safety information, standard procedures, safety training, incident reporting, research, safety inspections, etc., is imperative. Furthermore, risk assessment and management itself is a constituent of other factors like permit issuance, change management, and maintenance and repair. To provide complete protection against hazards and risks in the work environment, this process must be carried out with precision and attention to all factors related to safety culture.

Improving safety culture can be achieved by prioritizing factors with a higher impact score, as suggested by the study results. Nevertheless, it is important to remember that all the mentioned factors are significant and should not be disregarded.

In this study, we have used the term 'process safety culture' interchangeably with 'safety culture.' Given that the identified factors exist in all industries and work environments, these factors are generalizable and applicable to safety culture and other environments as well.

## Conclusion

In this research, a comprehensive analysis strategy has been introduced to examine the influential factors of process safety culture. By integrating hidden content analysis, DEMATEL, and fuzzy sets techniques, a robust and quantitative assessment of these factors has been achieved, leading to valuable insights on how to improve process safety culture. The key contributions and findings of this study are noteworthy:

i. Qualitative results: Through a combination of literature review and the Delphi technique, a comprehensive set of influential factors of process safety culture was identified.
ii. Quantification of qualitative results: By combining hidden content analysis with fuzzy DEMATEL, the qualitative results obtained from hidden content analysis were quantified. This conversion of linguistic estimates into fuzzy numbers allowed for a more precise and reliable analysis, reducing ambiguity in expert judgments.
iii. Identification of cause-effect relationships: The proposed approach facilitated the identification of cause-effect relationships among the different factors involved in creating process safety culture. Through the fuzzy DEMATEL technique, influential factors and their interrelationships were determined, providing deeper insights into the causality of process safety culture.
iv. Improved analysis reliability: By utilizing fuzzy sets, the analysis achieved higher levels of accuracy and reliability in assessing the impact of various factors on creating process safety culture. The fuzzy DEMATEL method enhanced the robustness of the results, enabling organizations to make informed decisions based on more reliable data.
v. Enhanced improvement of process safety culture: The integration of hidden content analysis and fuzzy DEMATEL allowed organizations to identify and prioritize the main influential factors closely related to safety culture. By addressing these critical factors, organizations can effectively reduce barriers and enhance required actions, leading to an improved process safety culture.

In conclusion, the developed strategy combining hidden content analysis, DEMATEL, and fuzzy sets proves to be a valuable and effective approach for analyzing influential factors of process safety culture and enhancing it in process industries. The ability to quantify qualitative data, identify cause-effect relationships, and prioritize influential factors provides a comprehensive and actionable understanding of the causality of process safety culture. By adopting this approach, industries can proactively address vulnerabilities, mitigate barriers, and continuously improve their safety culture.

### Limitations of the study

In this study, an attempt has been made to identify a comprehensive set of influential factors. However, due to the complex nature of safety culture and process industries, there may be other influential factors as well. As well, potential biases in expert opinions can be mentioned as study limitations.

## Data Availability

The datasets used and/or analyzed during the current study are available from the corresponding author upon reasonable request.
